# Water accelerated self-healing of hydrophobic copolymers

**DOI:** 10.1038/s41467-020-19405-5

**Published:** 2020-11-12

**Authors:** Dmitriy Davydovich, Marek W. Urban

**Affiliations:** Department of Materials Science and Engineering, Clemson University, Clemson, SC 29634 USA

**Keywords:** Polymers, Polymer characterization, Polymers

## Abstract

Previous studies have shown that copolymer compositions can significantly impact self-healing properties. This was accomplished by enhancement of van der Waals (vdW) forces which facilitate self-healing in relatively narrow copolymer compositional range. In this work we report the acceleration of self-healing in alternating/random hydrophobic acrylic-based copolymers in the presence of confined water molecules. Under these conditions competing vdW interactions do not allow H_2_O-diester H-bonding, thus forcing nBA side groups to adapt L-shape conformations, generating stronger dipole-dipole interactions resulting in shorter inter-chain distances compared to ‘key-and-lock’ associations without water. The perturbation of vdW forces upon mechanical damage in the presence of controllable amount of confined water is energetically unfavorable leading the enhancement of self-healing efficiency of hydrophobic copolymers by a factor of three. The concept may be applicable to other self-healing mechanisms involving reversible covalent bonding, supramolecular chemistry, or polymers with phase-separated morphologies.

## Abstract (web summary)

Self-healing of polymers became a vivid research area, but self-healing under water and its mechanistic concepts are less investigated. Here, the authors report water accelerated self-healing in a pMMA/nBA copolymer and demonstrate that perturbation of ubiquitous van der Walls forces upon mechanical damage in hydrophobic polymers in the presence of water is energetically unfavorable and accelerates self-healing.

## Introduction

Placing monomer units in an orderly fashion into a macromolecule may facilitate self-healing because upon mechanical damage, neighboring polymer chains return to their original conformations due to enhanced van der Waals interactions^1^. This approach is advantageous because it eliminates chemical and physical alterations and enables multiple recovery of thermoplastic polymers upon mechanical damage, thus expanding their functionality and sustainability. Obtaining materials with a longer life span also requires consideration of external environments to which polymers are exposed, for example, water. Hydrophobic nature of the majority of polymers though suggests that the presence of hydrophilic water should not impact self-healing properties. For that reason, to achieve water-induced self-healing, multilayered polyelectrolytes^2^ and redox-switchable supramolecular^3^ were proposed or sugar moieties^4^ incorporated into polymer networks. Considering that the hydrophobic effect is critical in many diverse phenomena, from the cleaning of laundry to emulsion synthesis or the assembly of proteins into functional complexes, theoretical studies^5^ have taught us that this typically multifaced effect depends on whether hydrophobic molecules are individually isolated or reassembled into larger hydrophobic structures. For example, water molecules can readily participate in four H bonds with a single methane molecule, but in larger hydrophobic aggregates, such as polymers, hydration of water is significantly diminished^6^. Here, we show that if a polymer is physically damaged resulting in a chain separation, water molecules may disrupt vdW interactions and participate in self-H-bonding, thus affecting self-healing. When mechanical load is removed, unfavorable polymer–water interactions within hydrophobic domains will lead to the expulsion of water from the system and rapid regeneration of polymer–polymer interactions due to enhanced interchain cohesive energies, thus leading to potentially faster self-repair.

## Results

**Fig. 1:**
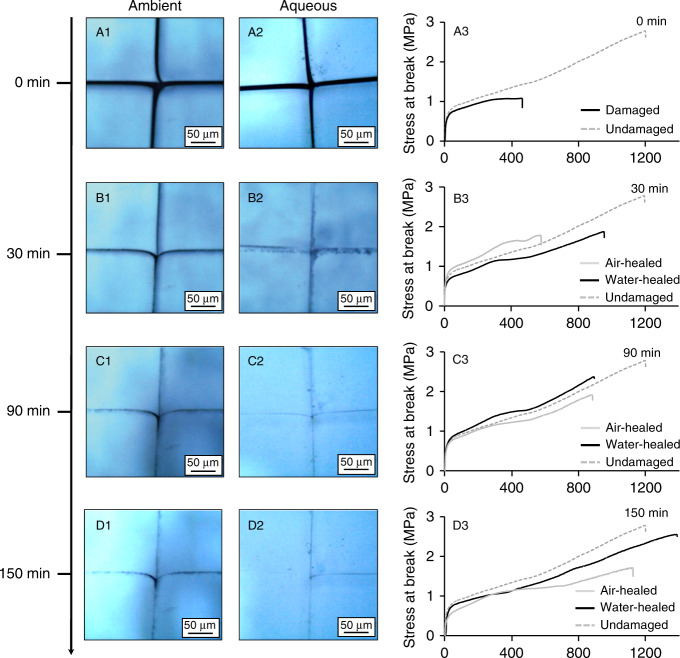
Optical images of air-cut/air-healed and air cut/water-healed p(MMA/nBA) copolymers as a function of time and the corresponding tensile analysis. Self-healing of 50/50 p(MMA/nBA) copolymers damaged in air and healed in ambient (A1–D1) and aqueous (A2–D2) environments. Tensile analysis of undamaged/damaged cycle (A3) and air/water-healed copolymer films at 30 (B3), 90 (C3), and 150 (D3) min.

We examined self-healing properties of self-healable poly(methyl methacrylate/n-butyl acrylate) [p(MMA/nBA)] copolymers composed of 50/50 MMA/nBA monomer molar ratio forming “key-and-lock” interactions in the presence and absence of water. Figure 1 illustrates optical images of air-cut/air-healed (A1–D1) and air-cut/water-healed (A2–D2) copolymers as a function of time. It turns out that the presence of aqueous environments during autonomous self-healing results in significantly faster (~3×) self-healing (A2–D2). Tensile analysis before damage and after self-repair under the same conditions (Fig. 1, A3 to D3) revealed that water-healed copolymers also exhibit faster tensile recoveries. After 30 min (Fig. 1, B3) air-cut/water-healed copolymers recover ~80% of stress at break (δ_break_) and maximum elongation (ε_max_), whereas during the same time, air-healed copolymers recover 70 and 46% of their respective values. After 150 min, air-cut/water-healed copolymers (Fig. 1, D2 to D3) recover ~100% of δ_break_ and ε_max_ values, whereas air-healed counterparts recover 87 and 83% (Supplementary Table 1), respectively. A summary of copolymer properties is provided in Supplementary Table 1.

**Fig. 2:**
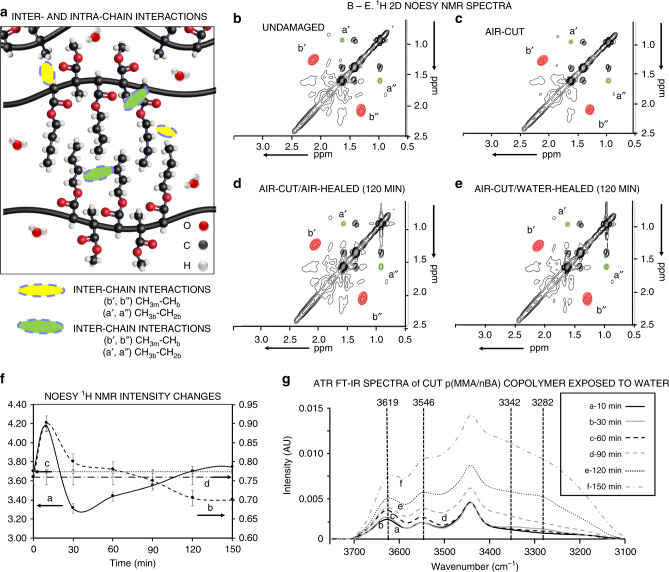
Anticipated inter- and intra-chain interactions supported by through-space two-dimensional NMR measurements and ATR FT-IR analysis. **a** Inter- and intrachain interactions due to side groups and copolymer backbone in p(MMA/nBA); inter-/intra-CH_3b_–CH_2b_ are represented by a′ and a″ resonances at 1.62 and 0.96 ppm, respectively; inter-/intra-CH_3m_–CH_b_ are represented by b′ and b″ resonances at 2.11 and 1.28 ppm (intensity changes of a′, a″, b′, and b″ resonances in NMR experiments are listed in Supplementary Table 4). 2D NOESY ^1^H NMR spectra of p(MMA/nBA: **b** undamaged, **c** air-cut/air-healed (0 min); **d** air-cut/air-healed (120 min); **e** air-cut/water-healed (120 min); **f** NOESY cross-peak intensities of CH_3m_–CH_b_ (a′, a″) (curve a) CH_3b_–CH_2b_ (b′, b″) (curve b), and undamaged (curve c) resonances as a function of time for air-cut/water-healed specimens. All resonances were normalized to α-methyl-CH_3_ and the -OCH_3_ proton cross-peaks at 3.61, 0.87 ppm (not shown) due to MMA units. **g** ATR-FTIR spectra of damaged p(MMA/nBA) copolymer exposed to water for 0 (a), 30 (b), 60 (c), 90 (d), 120 (e), and 150 (f) min.

To elucidate the role of water molecules on accelerated damage-repair cycles in hydrophobic copolymers, we considered inter- and intrachain interactions of CH, CH_2_, and CH_3_ groups along the copolymer backbone and side chains (Fig. 2a). If water molecules alter van der Waals (vdW) hydrophobic interactions, of primary interest are through-space CH_3b_–CH_2b_ and CH_3m_–CH_b_ as well as through-bond interactions in the presence and absence of water. Using nuclear Overhauser effect spectroscopy (NOESY)^7^ and correlation spectroscopy (COSY) 2D ^1^H NMR spectroscopy, we analyzed resonances at 1.62/0.96 (a′, a″) and at 2.11/1.28 (b′, b″) ppm due to through-space CH_3b_–CH_2b_ and CH_3m_–CH_b_ interactions for undamaged (Fig. 2b), air-cut (Fig. 2c), air-cut/air-healed (Fig 2d), and air-cut/water-healed (Fig. 2e) copolymers. The intensities of CH_3m_–CH_b_ (Fig. 2f, curve a) and CH_3b_–CH_2b_ (Fig. 2f, curve b) resonances initially increase, and as self-healing progresses, they diminish at different rates; the CH_3m_–CH_b_ (curve a) resonance decreases faster, but at the initial stages of self-healing increases to level off after 120 min; the CH_3b_–CH_2b_ (curve b) resonance continually decreases to level off upon self-healing. The initial increase of both resonances is attributed to shorter through-space distances of the backbone and side groups likely resulting from the compression of neighboring copolymer chains during mechanical damage, which are gradually restored to their original state. These results are further supported by a series of NOESY and COSY ^1^H NMR experiments (Supplementary Figs. 1 and 2 and Supplementary Tables 2 and 3), indicating that the presence of water forces chains into closer proximity after mechanical damage, thereby decreasing the time necessary for repair, but longer H_2_O exposure times diminish interchain side- group interactions, likely due to H-bonding in the proximity of hydrophilic esters of MMA and nBA side-group acceptors of polar water molecules. The values for undamaged copolymers do not change (Fig. 2f, curve c), and the intensities of a′, a″, b′, and b″ resonances for all NMR experiments are shown in Supplementary Table 4.

Because water may exist in the form of single molecules, dimers, or small and large clusters^8,9^, we measured ATR-FTIR spectra of mechanically damaged p(MMA/nBA) copolymer exposed to water for 0 (a), 30 (b), 60 (c), 90 (d), 120 (e), and 150 min (f) (Fig. 2g). Relative ATR FTIR band intensity changes at 3619, 3546 cm, 3342, and 3282 cm^−1^ attributed to free and dimerized water as well as small (3, 4) and large water clusters (>4)^9,10^, respectively, indicate that as the exposure time to water increases during self-healing, the fraction of free water diminishes, whereas dimerized content remains relatively constant (Supplementary Table 5). As expected, the amount of small and large clusters increases at longer exposure times. Carbonyl groups of esters practically do not form C=O….H_2_O H-bonding as shown by unchanged C=O vibrations at 1728^−1^ as a function of exposure to H_2_O. Only minute amounts of H-bonding are displayed by a slight broadening around 1710 cm^−1^, which parallels the increase of OH-bending vibrations at 1643 cm^−1^ (Supplementary Fig. 5).

Although ATR-FTIR analysis indicates that the type of water associations in close proximity of polymer chains plays a significant role in self-healing, to further elucidate molecular origins of enhanced self-healing in water, we utilized MD simulations (MD) in the presence and absence of water, which were employed under isothermal (NVT) and isoenergetic equilibration (NVE) conditions. We varied H_2_O to MMA/nBA-repeating unit ratios (R_w_ = H_2_O:MMA/nBA) from 1:4, 1:2, 3:4, 1:1, 3:2, to 2:1, which correspond to 105, 210, 315, 420, 630, and 840 water molecules per 420 copolymer MMA/nBA units. Table 1 summarizes MD results and shows that the total cohesive energy (CE_total_) and cohesive energy due to H-bonding (CE_H_) increases as the number of H_2_O molecules increases; cohesive energy due to vdW (CE_vdW_) forces reaches maximum when *R*_w_ = 1:1. The same behavior is observed for the radius of gyration (*R*_g_) and end-to-end distances (*r*_eq_). This behavior is not observed for non-self-healable p(MMA/nBA) copolymers with 60/40 and 40/60 MMA/nBA molar ratios (Supplementary Tables 6 and 7).

**Table 1:** Total cohesive energy (CE_total_), vdW cohesive energy (CE_vdW_), hydrogen-bonding cohesive energy (CE_H_), total cohesive energy density (CED_total_), vdW cohesive energy density (CED_vdW_), hydrogen-bonding cohesive energy density (CED_H_), radius of gyration (*R*_g_), and average chain end-to-end distance (*r*_eq_) as a function of # of H_2_O molecules in 50/50 p(MMA/nBA) copolymer (*R*_w_ is the ratio of the # of H_2_O to # of MMA/nBA-repeating units in p(MMA/nBA)).

# of H_2_O molecules	*R*_w_	CE_total_	CE_vdw_	CE_H_	CED_total_	CED_vdW_	CED_H_	*R*_g_(Å)	*r*_eq_(Å)
		kJ × 10^3^	kJ × 10^3^	kJ × 10^3^	kJ × 10^5^/m^3^	kJ × 10^5^/m^3^	kJ × 10^5^/m^3^		
0	0:1	8.73	8.32	0	2.00	1.98	0	15.5	34.0
105	1:4	8.20	7.49	0.72	1.86	1.70	0.16	15.1	27.2
210	1:2	8.87	7.41	1.56	1.92	1.59	0.33	14.2	25.3
315	3:4	10.20	7.61	2.48	2.12	1.61	0.51	14.9	30.3
420	1:1	11.28	7.70	3.57	2.28	1.56	0.72	15.8	41.9
630	3:2	12.56	7.20	5.36	2.39	1.37	1.02	16.1	38.7
735	7:4	13.26	6.39	6.87	2.44	1.18	1.26	15.1	27.1
840	2:1	12.29	4.19	8.08	2.17	0.78	1.39	14.7	29.3

**Fig. 3:**
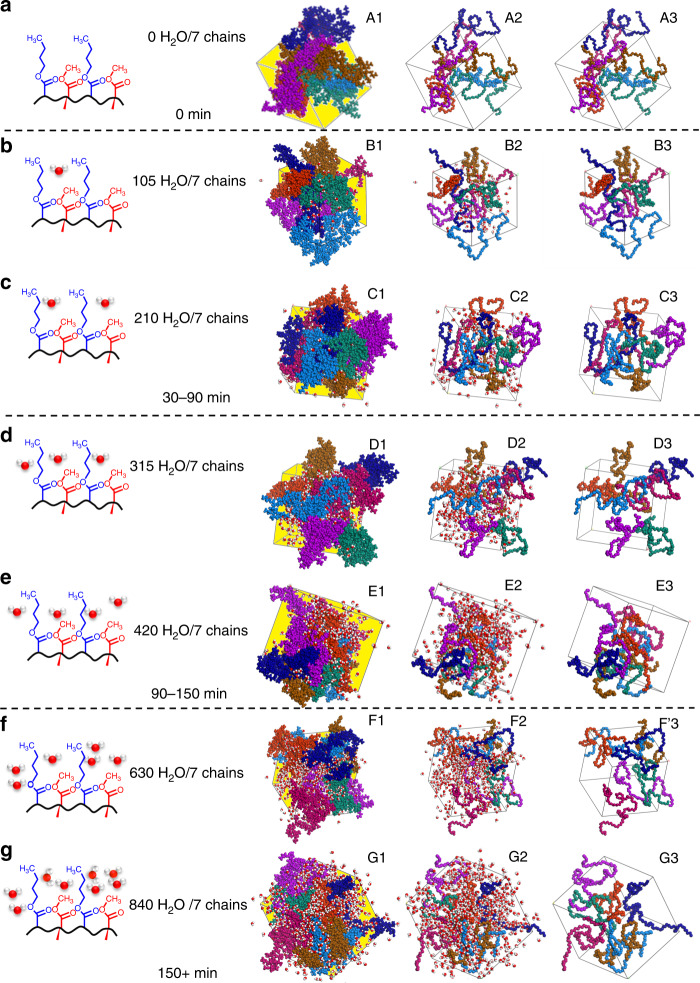
The results and analysis of MD simmulations in the presence of variable amounts of water. **a**–**g** p(MMA/nBA) copolymers surrounded by H_2_O molecules (*R*_w_ = 0, 1:4, 1:2, 3:4, 1:1, 4:3, and 2:1); A1/A2/A3–G1/G2/G3 illustrates the results of MD simulations: A1–G1–A3–G3 are image-extracted macromolecules from MD simulations: A1–G1 are copolymer backbone, side groups, and water, A2–G2 are backbone atoms and water, and A3–G3 are backbone units.

When *R*_w_ is ≤1:2, the CE_vdW_ values decrease from 8.32 × 10^3^ for water-free p(MMA/nBA) chains to 7.41 × 10^3^ kJ; the end-to-end distances (*r*_eq_) also decrease as chains begin to assume globular conformations (*r*_eq_ ≤ 32 Å) from the initial extended helical state *r*_eq_ = 34 Å, thus further resulting in smaller CE_vdW_ values. For *R*_w_ = 1:1, the interchain CE_vdW_ values increase to 7.70 × 10^3^ kJ as the chains return to the extended helical states (*r*_eq_ = 41.9 Å). The *R*_g_ also decreases from 15.5 Å to 14.2 Å when *R*_w_ = 1:4–3:4, but the *R*_g_ values return to the initial values when *R*_w_ = 1:1. When *R*_w_ is > 1:1, interchain vdW forces are weaker and chains transition from extended–helical to globular conformations with increased interchain distances. This is reflected by the decrease of CE_vdW_ and *r*_eq_ values (Table 1). These data indicate that one H_2_O molecule per MMA/nBA-repeating unit will favor faster self-healing compared to air-cut/air-healed copolymers. This is illustrated in Fig. 3, A1–G1, A2–G2, and A3–G3 that shows the entire cell (A1–G1), along with the extracted backbones (A2–G2) and without (A3–G3) the surrounding water molecules. For an equilibrated unit cell without water (Fig. 3 A1–A3; *R*_w_ = 0), close interchain packing and yellow- shaded unoccupied space are the same as for *R*_w_ = 1:1 (one H_2_O molecule per MMA/nBA- repeating unit (Fig. 3, E1) cell). These results are in agreement with experimental NMR (Fig. 2b, Supplementary Figs. 1–4) and IR (Fig. 2e and Supplementary Table 5) analyses. For example, NOESY 2D ^1^H NMR data show that for non-self-healable p(MMA/nBA) copolymers with MMA/nBA molar ratios of 60/40 and 40/60 (Supplementary Fig. 4), nBA-rich (40/60) copolymer resonances due to CH_3b_–CH_2b_ (a′, a″) and CH_3m_–CH_b_ (b′, b″) interactions diminish upon mechanical damage (Supplementary Table 4), indicating that copolymer chains are further apart, but are enhanced for self-healable (50/50 compositions (Fig. 2b/B1 and c/C1)). For MMA-rich (60/40), the cross-peak intensities of (a′, a″) and (b′, b″) resonances are very weak, indicating fewer CH_3b_–CH_2b_ and CH_3m_–CH_b_ interactions. Although ideally to achieve self-healing only alternating copolymer topologies would be desirable, reactivity ratios of MMA (~2.9) and nBA (~0.3) inhibit their formation. However, for self-healable p(MMA/nBA) copolymer with MMA/nBA monomer molar ratio of 50/50, the increase of alternating MMA/nBA units to 37% (Supplementary Table 8) increases the CE_vdW_ values (8.45 × 10^3^ kJ) compared to non-self-healable MMA- and nBA-rich copolymers with 60/40 and 40/60 MMA/nBA monomer molar ratios.

**Fig. 4:**
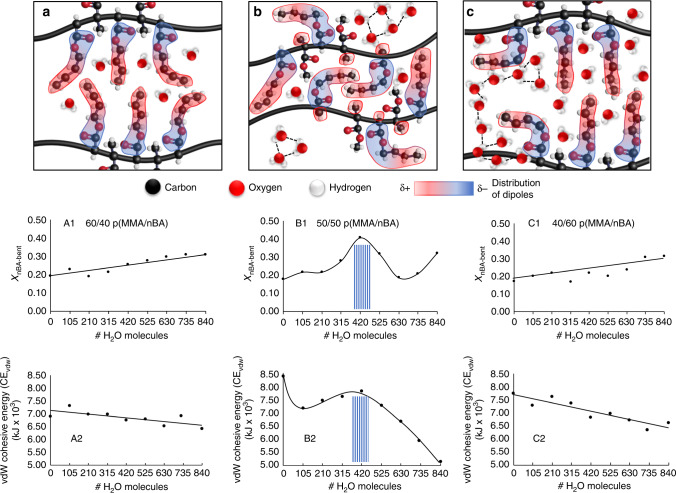
Visual representation of L-shape conformational changes in n-BA units resulting from interactions with H2O supported by experiments and MD simulations. Graphical depiction of interchain vdW interactions between neighboring p(MMA/nBA) copolymer chains containing 210 (**a**—H_2_O:MMA/nBA = 1:2), 420 (**b**—H_2_O:MMA/nBA = 1:1), and 840 (**c**—H_2_O:MMA/nBA = 2:1) water molecules. A fraction of bent nBA side groups (X_nBA-L-shaoe_) plotted as a function of # H_2_O molecules for non-self-healable 60/40 p(MMA/nBA) (A1), self-healable 50/50 p(MMA/nBA) (B1), and non-self-healable 40/60 p(MMA/nBA) (C1). VdW cohesive energy plotted as a function of # H_2_O molecules for non-self-healable 60/40 p(MMA/nBA) (A2), self-healable 50/50 p(MMA/nBA) (B2), and non-self-healable 40/60 p(MMA/nBA) (C2).

If one H_2_O molecule per (MMA/nBA) repeat unit accelerates self-healing of a hydrophobic copolymer, the question is what inter- and intrachain interactions are responsible for this behavior. Taking advantage of the ability of MD simulations to visually assess conformational changes, p(MMA/nBA) copolymer chains with *R*_w_ = 1:2, 1:1, and 2:1 were extracted and analyzed. While Supplementary Information (Supplementary Fig. 11) provides further details, when *R*_w_ = 1:1 (Fig. 4b), not only interchain distances are smaller (Table 1), but a larger fraction (~42%) of nBA side groups takes L-shape conformations compared to 1:2 (Fig. 4a) and 2:1 (Fig. 4c) H_2_O:MMA/nBA ratios (Supplementary Table 9). Molar fraction of L-shaped nBA side groups and CE_vdW_ changes plotted as a function of # of H_2_O molecules in p(MMA/nBA) copolymers with 60/40 (Fig. 4A1), 50/50 (Fig. 4B1), and 40/60 (Fig. 4C1) monomer molar ratios shows that the highest fraction of L-shaped nBA side groups coincides with the maximum of CE_vdW_ values observed for 50/50 composition when *R*_w_ = 1:1 (Fig. 4b–B2). When approaching *R*_w_ = 1, H_2_O molecules begin to form small clusters between the chains, resulting in self-H-bonding that causes conformational changes of nBA enhancing vdW interaction and accelerated self-healing. The same phenomenon of accelerated self-healing in the presence of H_2_O was observed for even more hydrophobic poly(methyl methacrylate/n-pentyl acrylate) [p(MMA/nBA)] copolymer with a 50/50 MMA/nPA molar ratio in p(MMA/nPA) (Supplementary Fig. 6, air-cut/air-healed (A1–A5) and air-cut/water-healed films (B1–B5)).

Considering directionality and polarity differences between H-bonding and vdW interactions, the former facilitates localized bonding directionality and polarity (hydrophilicity). In contrast, vdW interactions offer high nondirectional polarizability (hydrophobicity) that can be enhanced by closer proximity of neighboring chains^11^. For predominantly alternating/random hydrophobic p(MMA/nBA) copolymer topologies forming helix-like conformations, this was accomplished by interdigitated “key-and-lock” associations of neighboring chains. The presence of one H_2_O molecule per one copolymer-repeating unit (MMA/nBA) strengthens interchain forces by forcing a significant fraction of the nBA side groups to take L-shape conformations, thus resulting in stronger dipole–dipole forces and the decreased interchain distances. This environment creates a viscoelastic response that not only energetically favors self-repair upon chain separation, but also accelerates this process due to closer proximity of the neighboring chains and the ability to rapidly restore initial conformations. Unlike one type of interaction (H-bonding, coordination chemistry, covalent rebonding, and others^12^) that is typically introduced to achieve self-healing properties, the presence of confined H_2_O molecules in the proximity of ester groups may generate local plasticizing effect due to H-bonding that increases vdW interactions of nBA side groups. Although plasticizing effects are well-documented for hydrophilic polymers^13,14^, perturbation of vdW forces upon mechanical damage in the presence of controllable amount of water is energetically unfavorable and accelerates self-healing in hydrophobic copolymers.

## Methods

### Materials

Methyl methacrylate (MMA), n-butyl acrylate (nBA), chloroform, methyl ethyl ketone (MEK), and initiator azobisisobutyronitrile (AIBN) were purchased from Sigma-Aldrich and used as received. Hexane and tetrahydrofuran (THF) were purchased from Thermo Fisher-Scientific and used as received. Chloroform-D was purchased from ACROS Organics.

### Copolymerization

Poly(methyl methacrylate/n-butyl acrylate) [p(MMA/nBA)] copolymers with 40/60, 50/50, and 60/40 MMA/nBA molar ratios were synthesized using solution polymerization in THF. In a typical synthesis, a total of 0.042 moles of the monomer was dissolved in 5 ml of THF, mixed with 0.5% w/w of AIBN, and purged with N_2_ for 30 min. The air-free system was placed in a heat bath at 75 °C and allowed to react for 8 h. The resulting product was precipitated in hexane and redissolved in THF (300 mg/ml) prior to film formation. The same method was utilized to copolymerize poly(methyl methacrylate/n-pental acrylate) [p(MMA/nPA)]. The properties of p(MMA/nBA) and p(MMA/nPA) are summarized in Supplementary Table 1.

### Copolymer analysis and characterization

Gel permeation chromatography (GPC) was performed using Waters GPC and calibrated with GPC- grade polystyrene standards using the refractive index (RI) detector. The copolymers were dissolved in HPLC-grade chloroform (Sigma-Aldrich) and passed through a 0.2-µm filter prior to each measurement.

Differential scanning calorimetry (DSC) measurements were conducted using Q 100 series TA Instrument. The heating rate of 20 °C/min in the −100 to 100 °C range was used. Data analysis was performed using TA Universal Analysis software. The glass transition (*T*_g_) of the 50/50 p(MMA/nBA) copolymer was 7 °C, whereas for 40/60 and 60/40, −5 and 28 °C.

Tensile analysis was conducted using Instron Model 5500 R 1125. Stress–strain curves were obtained for undamaged and air-cut/air-healed (3 h) and air-cut/water-healed (3 h) copolymers. The gauge length and the strain rate were set to 1.0 cm and 40 mm/min, respectively. The average thickness of the films was 0.5 mm. To determine the self-repairing capability of p(MMA/nBA) copolymers, 3 × 1 × 0.025-cm films were damaged in air using a stainless-steel razorblade to obtain cuts with a width of 20 μm and a depth of 50 μm. These films were allowed to heal under ambient and aqueous conditions at 25 °C for up to 150 min. Tensile stress–strain properties of the damaged–healed films were recorded at 30-min intervals.

Thermogravimetric analysis (TGA) was conducted using a TA Instrument Hi-Res TGA 2950. Prior to analysis, copolymer films were cut into 3 × 3-mm squares with a thickness of 200–300 μm and immersed in deionized (D)I H¬2 O for 0, 30, 60, 90, 120, 150, and 1440 min (24 h). A typical TGA experiment was carried out from 25 to 150 °C at a heating rate of 20 °C/min. The resulting data were processed using TA Universal Analysis vs. 5.4 software.

Solution ^1^H NMR measurements were performed using a 500-MHz Bruker Avance spectrometer. A standard concentration of 5.0 mg/ml of copolymer was dissolved in chloroform-D, and in a typical experiment, 64 scans were collected. All spectra were processed using MestReNova software.

Nuclear Overhauser effect spectroscopy (NOESY) spectra were acquired on NEO Avance 500-MHz Bruker spectrometer. Prior to analysis, copolymer 0.5 × 0.5 × 0.025-cm films were cut using a razorblade grid, resulting in 441 pieces. Such prepared specimen was placed into CDCl_3_ at a concentration of 15 mg/ml and allowed to dissolve without agitation for 10 min prior to analysis. The relaxation and mixing times were optimized and set to 1.5 and 0.6 s, respectively. Eight consecutive scans were co-added to generate NOESY spectra. Reproducibility of NMR experiments was determined by conducting multiple damage-repair cycle experiments using different damaged sections of copolymer films. The analysis of NOE intensities and chemical shifts due to ^1^H resonances agree with the literature data^15–20^.

Attenuated total reflectance (ATR) Fourier transform-infrared (FTIR) analysis was conducted using Agilent Carry 680 μATR-FTIR single-beam spectrometer set at 4 cm^−1^ resolution with 64 scans/spectrum (19). To analyze the water content of a damaged copolymer as a function of exposure time, 50/50 p(MMA/nBA) 0.5 × 0.5 × 0.03-cm films were damaged (20 vertical and horizontal cuts ~30–50 μm in depth) and were allowed to heal in water.

Dynamic light-scattering (DLS) experiments were conducted on Zetasizer Nano-SZ Zen3600 manufactured by Malvern Instruments. The instrument library was used to identify solvents and p(MMA) standard.

Molecular dynamic (MD) simulations were conducted using Accelrys (BIOVIA) Materials Studio vs 5.5. Prior to simulations, seven identical chains with sixty number average of repeating units (X_n_) were placed within a unit cell at a density of 1.125 g/cm^3^ and geometrically optimized using the Dreiding Forcefield. Each cell was filled with a discrete amount of water molecules to mimic different degrees of solvation with the repeating copolymer unit to water ratios (*R*_w_) of 1:4, 1:2, 1:1, 3:4, and 2:1. To simulate an unperturbed copolymer state, initial MD NVT simulation was conducted at 298 K for 100 ps at a time step of 0.33 fs using Berendsen thermostat and the previously selected Dreiding Forcefield. The CED, CE_H_, CE_vdW_, and *R*_g_ values were calculated using the Forcite module. The Dreiding Forcefield was utilized with the build-in TIP3P water model. Due to the fact that each chain consisted of 60 repeating units, access of one monomer at the expense of another one results in changes in MMA/nBA molar ratio by two. Thus, MD simulations of p(MMA/nBA) copolymers were conducted on 50/50, 60/40, and 40/60 molar ratios. All data are available from the authors upon reasonable request.

## Supplementary information

Supplementary Information

## Data Availability

All data needed to evaluate the conclusions in the paper are present in the paper or the Supplementary Information.
