# Supplementary material for: Water accelerated self-healing of hydrophobic copolymers
Source: Nat Commun. 2020 Nov 12;11:5743. doi: 10.1038/s41467-020-19405-5 (PMC7665198; doi:10.1038/s41467-020-19405-5)
Supplement: Supplementary file 1 — Supplementary Information [file 41467_2020_19405_MOESM1_ESM.pdf]

Supplementary Information

Water Accelerated Self-Healing of Hydrophobic Copolymers

Dmitriy Davydovich and Marek W. Urban*
Department of Materials Science and Engineering
Clemson University, Clemson, SC 29634
correspondence to: mareku@clemson.edu

*- mareku@clemson.edu

Supplementary Discussion

Supplementary Table 1 summarized properties of p(MMA/nBA) and p(MMA/nPA) copolymers. To remove water, each specimen was placed under vacuum (0.08 atm) for 30 min at 25 °C. Stress at break and max strain measurements are summarized in Supplementary Table 2.

Supplementary Figure 2 illustrates the changes in the CH_{3b}-CH_{2b} and CH_{3m}-CH_b cross-peak NOE intensity as a function of time for air-cut/air-healed and air-cut/water-healed films. These data indicate that unlike in water-healed specimens discussed in Main Docs., air-cut/air-healed films regenerate the side group CH_{3b}-CH_{2b} interactions within 90 minutes, but are unable to recover backbone CH_{3m}-CH_b interactions in 150 min. Longer recovery of the backbone interactions elucidates the longer repair times for air-cut/air-healed copolymers.

In order to determine the effect of water and agitation by vertexing on copolymers, three independent experiments on undamaged 50/50 p(MMA/nBA) films exposed to water for 1 and 14 days as well as on copolymer solutions ($CDCl_3$) vortexed for 10 min were conducted. NOESY spectra of undamaged 50/50 p(MMA/nBA) after 1 day (A), 14 days (B) in water, and vortexed copolymer (C) without water exposure (Supplementary Figure 3) show that exposure to water for 1 day diminishes both side group CH_{3b}-CH_{2b} (a', a'') and backbone CH_{3m}-CH_b (b', b'') interactions. Notably, the side group cross-peak intensities decrease to the original value (0.71) in air-cut/air-healed copolymers after 120min (C). As the exposure time is extended to 14 days (B), while backbone cross-peak interactions show a small increase, the side-group interactions continue to diminish, suggesting that prolonged exposure to H_2O decreases inter-chain side group vdW interactions, likely by the formation of H-bonding with the ester moieties. As seen, undamaged copolymers agitated by vertexing (Supplementary Figure 2-C), exhibit the lowest NOE intensities for both side group and backbone interactions, due to complete solubilization of chains and dissociation of inter-chain interactions.

For comparison, Supplementary Figure 4 illustrates NOESY spectra of undamaged (A) and damaged (A' - 0 min) 40/60 p(MMA/nBA) copolymers. Undamaged (B) and damaged (B' – 0 min) NOESY spectra for 60/40 p(MMA/nBA) copolymers are shown in Figure 4. The decrease of resonances a', a'', b', and b'' in non-healable 42/58 and 63/47 p(MMA/nBA) copolymer compositions (Supplementary Figure 3) do not undergo initial chain compression during mechanical damage. Also, MMA-rich 60/40 p(MMA/nBA) copolymer exhibits low NOE crosspeak signals due to a lack of the vdW interactions between both the side groups and backbones.

ATR FT-IR band intensities of the 3619 and 3546 cm^{-1} bands due to "free" and dimer water as well as the bands at 3342 and 3282 cm^{-1} attributed to small and large water clusters (20) were normalized to the alkane stretching band at 2932 cm^{-1}. Contributions of free, dimer, small cluster and large cluster water molecules were calculated by the ratio of respective band intensity to the total amount of water represented by the sum of all band intensities. Supplementary Table 5 summarizes the fraction of water molecules due to free ($\chi_{\text{Free H2O}}$), dimer ($\chi_{\text{Dimer H2O}}$), small cluster ($\chi_{\text{Small cluster H2O}}$), and large cluster ($\chi_{\text{Large cluster H2O}}$) states as a function of time. Supplementary Figure 5 illustrates unchanged carbonyl band intensities as a function of exposure to H_2O.

A series of control experiments were performed in an effort to assess the effect of water molecules on CE_{vdW} values for p(MMA) and p(nBA) homopolymers and H_2O and compared to self-healable 49/51 p(MMA/nBA) copolymer (Supplementary Figure 7). While p(MMA) and n(BA) homopolymers and H_2O values exhibit expected decrease with the increasing (except H_2O) content, self-healable p(MMA/nBA) shows the maximum at around 420 water molecules which corresponds to 1:1 molar ratio of MMA/nBA:H_2O (Supplementary Figure 4 D').

MD simulations allow visual assessment of inter-chain distances and orientation of nBA side groups. Previous studies showed that key-and-lock interdigitated copolymer chains are formed due to enhanced vdW interactions,(*1*) but the presence of H_2O molecules causes orientation

changes, primarily visible for nBA side groups which take L-shape conformations at the top of -OCH_2- moiety (nBA-L). To semi-quantitatively determine the percentage (%) of nBA-L groups as a function of # of H_2O molecules we counted nBA side groups above -OCH_2- carbon unit which were colinear or parallel to p(MMA/nBA) backbone. Supplementary Figure 8 illustrates that A and C conformations do not meet this criterion, whereas B and D contribute towards L-shaped n-BA side groups. The results are summarized in Supplementary Table 8 and shows that when approaching 420 H_2O molecules (R_w = 1:1), the molar fraction of bent nBA units (χ_{nBA-L}) reaches is the highest (~0.42). In the absence of H_2O the % of nBA-L shaped units ranges from 17-19% regardless of monomer 60/40, 50/50. 40/60 ratios.

In another MD simulation p(MMA/nBA) copolymers with 60/40 and 40/60 monomer molar ratios were used to determine how copolymer topology will influence CE_{vdw} as a function of # of H_2O molecules. For each copolymer composition two copolymer topologies were examined: 1) larger amounts of blocks with smaller sizes (min. 3 repeating units) and smaller amounts of blocks with larger sizes (> 5 repeating units). Supplementary Table 8 summarizes the CE_{vdw} values as a function of # H_2O molecules for 60/40, 50/50, and 40/60 copolymer compositions and show that fewer and smaller size blocks favor higher vdW energy.

Typical 2D NOESY/COSY ^{1}H NMR (*2, 3*) experiment takes ~80 min. Since p(MMA/nBA) copolymer is dispersed in $CDCl_3$ solvent, there is a possibility that the solvation process will distort inter-chain interactions. To determine if during NMR acquisitions severed copolymer films are not solubilized, we conducted dynamic light scattering (DLS) experiments in the same solvent used in NMR experiments as well as in methyl ethyl ketone (MEK). To mimic exact experimental conditions utilized in NMR experiments the same sample preparation (cutting into 441 pieces) was used. Supplementary Table 10 illustrates the results of DLS analysis for 50/50 p(MMA/nBA) films after 0, 30, 60 and 90 min in each solvent.(*4*) These data show that for undamaged specimens dissolved in $CDCl_3$ without agitation, two separate particle sizes of 289 and 10.5 nm are detected. After exposure to the solvent for extended periods of time (90 min) the particle sizes decrease to 232 and 7.0 nm. These results are in agreement with 2D NOESY ^{1}H NMR analysis which showed that undamaged specimens' resonances responsible for CH_{3m}-CH_b and CH_{3b}-CH_{2b} resonances remain constant (Figure 2D, curve c).

Number average molecular weight (M_n) of 50/50 p(MMA/nBA) copolymer is 39 kDA and for fully extended chains, the average chain length is ~80 nm. Thus, the larger particle size is attributed to undissolved polymer chains indicating that inter-chain interactions are present after 90 min of $CDCl_3$ solvent exposure. In contrast, copolymer films which were agitated (vortexed) for 10 min in $CDCl_3$ prior to analysis showed significantly reduced two particle sizes of 54.8 and 19.2 nm, thus indicating that without mechanical energy input copolymer chains are still in an undissolved state, thus securing inter-chain interactions. We conducted parallel experiments in MEK solvent which has significantly greater solvating power. After dissolving films without agitation, average particle sizes are 20 nm at 0 min and 14 nm after 90 min. The 14-20 nm particle size originates from collapsed/folded copolymer chains. This is shown in Supplementary Figure 9 which illustrates undamaged vortexed copolymer size distribution in $CDCl_3$ and in MEK solvents at 0 and 90 min. These results indicate that while $CDCl_3$ disperses 50/50 p(MMA/nBA) copolymer chains, it does not eliminate inter-chain interactions, thereby supporting the fact that 2D NOESY ^{1}H NMR experimental conditions allow us to measure through-space inter-chain interactions. Supplementary Figure 10 illustrates DLS results of A) non-vortexed and B) vortexed undamaged particles in $CDCl_3$ at 0, 30, 60 and 90 min after initial dissolution. Although a small fraction of copolymer chains is solubilized (~10 nm peak), the majority remain in much larger clusters.

Visual assessment of extracted squares (a-1, b-1, c-1, and d-1) for p(MMA/nBA) with 50/50 MMA/nBA molar ratio for R_w = 1:2 and 2:1 and shows that conformations of nBA side groups are not significantly affected. In contrast, when the molar ratio is Rw = 1:1, significantly great

percentage of bent nBA groups is observed. Supplementary Figure 11 illustrates extracted chains from MD simulations and relative amounts of bent and non-bent nBA side groups.

H_2O w/w % Content Comparison

In an effort to determine H_2O w/w % and its role on accelerated self-healing of hydrophobic copolymers, the results of MD simulations were compared with experimental ATR FT-IR and TGA measurements.

MD Simulations. Using MD simulations, the average distance of H_2O molecules from p(MMA/nBA) chains were determined using the following criteria:

a. w/w % of H_2O molecules located < 2.5 Å.
b. w/w % of H_2O molecules located between 2.5 A and 5 Å.
c. w/w % of H_2O molecules located > 5 Å.

This choice was dictated by considering the impact of the proximity of H_2O molecules on vdW inter-chain interactions. The theoretical values of w/w % amount of H_2O plotted as a function of the number of H_2O molecules for each range of distances shown in Supplementry Figure 12 - a illustrates that when Rw = 1:1 (420 H_2O molecules, or 1:1 H_2O:MMA/nBA ratio), the minimum is observed at approximately ~2 w/w % of H_2O which is in a close proximity of copolymer chains (< 2.5 Å). Extracted from MD simulations copolymer chains with water molecules close to copolymer chains (< 2.5 Å) are shown in Supplementary Figure 12, a'. As a higher content of water molecules is introduced (Rw = 1:2 and higher), H_2O molecules form dimmer and larger clusters (Supplementary Figure 12, b') which steadily increase to become phase separated (Supplementary Figure 12, c') from copolymer chains. In these simulations it was assumed that only water which is in close proximity of polymer chains Supplementary Figure 12, curve a) alters inter-chain vdW interactions to accelerate self-healing, whereas the curve b+c represents the summary of w/w % of H_2O molecules 2.5 – 5 and > 5 Å distances. Small and large clusters of H_2O form H-bonds which do not significantly interfere with inter-chain vdW interactions (although it may exert pressure on the system). These data are also supported by ATR FT-IR analysis (Main Doc.) that H_2O may exist in the form of single molecules, dimers, or small and large clusters.

ATR FT-IR Analysis. Using intensity of OH bending vibrations of water at 1643 cm^{-1} and C=O stretching vibrations of acrylic esters at 1726 cm^{-1}, concentration of water in 50/50 p(MMA/nBA) copolymer as a function of time was determined using the Beer-Lambert law (A = εbc; where: A is the absorbance, ε is the extinction coefficient, b is the path length, and c is the concentration. The literature values of ε for OH bending and C=O stretching vibrations of 21.8 and 300 (cm^{-1} x mol^{-1}), respectively, were used.(*5, 6*) Relative concentrations of H_2O w/w % are summarized in Supplementary Table 12. As expected, as exposure time to water increases, the w/w % of H_2O also increases to ~1.7 w/w % and begin to approach theoretical values predicted by MD simulations (~2 w/w %; 420 H_2O molecules, or 1:1 H_2O:MMA/nBA ratio). Experiments conducted on undamaged and damaged copolymers show the same H_2O w/w % and are expected to deviate from MD-based estimations because ATR FT-IR is a surface technique,(*7, 8*) whereas MD simulations were conducted on the entire cell.(*8*)

Thermogravimetric Analysis (TGA). The results of TGA are summarized in Supplementary Table 13 and the representative TGA scan illustrated in Supplementary Figure 13 show that ~1.2 w/w % of H_2O (1440 min/24hrs exposure) is released above 130^oC. TGA expriments conducted on undamaged and damaged films show no difference in H_2O w/w %. These data further substantiate conclusions that hydrophobic copolymers will hydrophilic H_2O only to a fraction of H_2O (~1.2 to 1.6 w/w %), and to effectively accelerate self-healing one H_2O molecules per one MMA/nBA repeating unit is required.

In summary, a comparison of water uptake in MD simulations, ATR-FT-IR analysis, and TGA show expected variations, being the highest in MD simulations (~2 w/w%) because water was distributed throughtout the entire volume, whereas empirically measured using ATR-FT-IR and TGA, the actual uptake is in the range of 1.2 – 1.7 w/w %.

Supplementary Figures

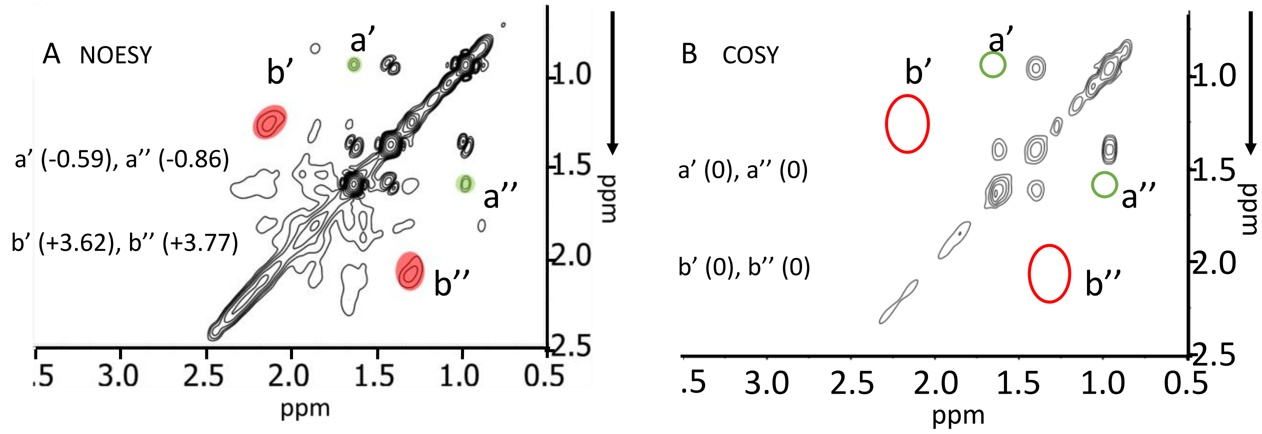

Supplementary Figure 1. NOESY (A) and COSY (B) ^{1}H NMR spectra of undamaged p(MMA/nBA) copolymers. Resonances a’, a”, b’, and b” in COSY spectra are not detectable.

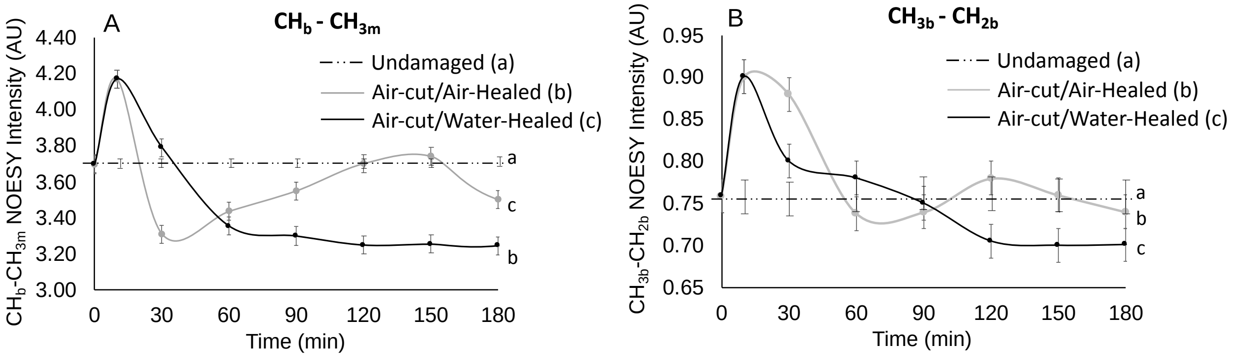

Supplementary Figure 2. CH_{3m}-CH_b (A) and CH_{3b}-CH_{2b} (B) NOESY cross-resonance intensities changes plotted as a function of time for undamaged (a), air-cut/air-healed (b), and air-cut/water-healed (c) copolymers. Controlled experiments for undamaged p(MMA/nBA) copolymers (curves a) as a function of time exhibit non-detectable intensity differences between in-air and in-water exposures. For much longer exposure times (days) there are significant changes.

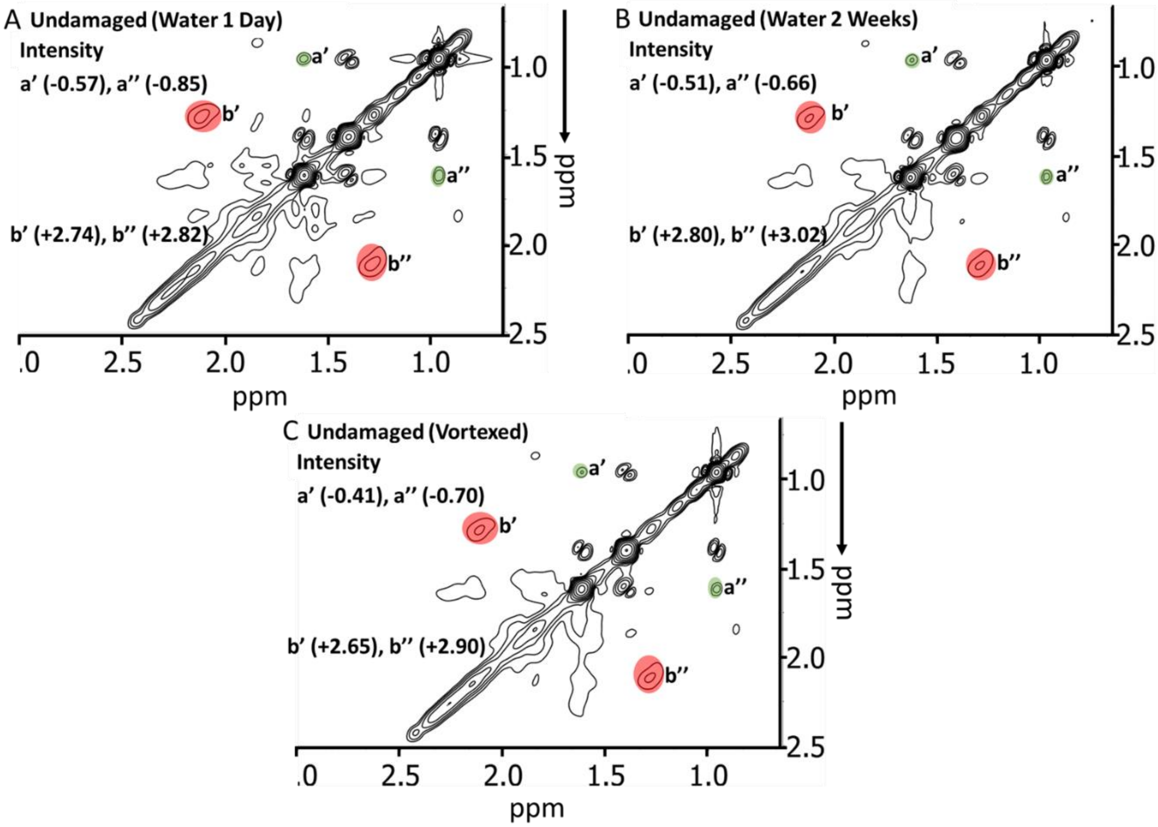

Supplementary Figure 3. ^{1}H NMR NOESY spectra of: A-undamaged 50/50 p(MMA/nBA) copolymer films after 1 day, B - two weeks of water exposure, and C - 50/50 p(MMA/nBA) copolymer films without water exposure, but after heavy agitation in $CDCl_3$.

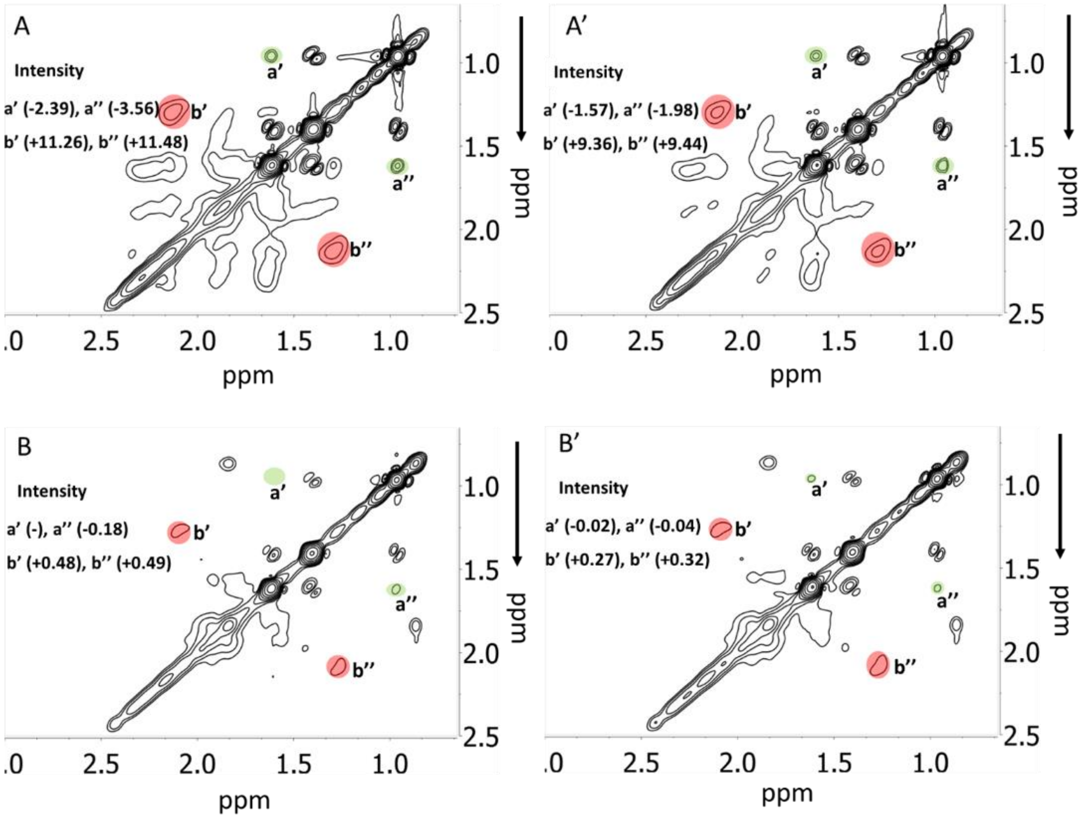

Supplementary Figure 4. ^{1}H NMR NOESY spectra of 40/60 p(MMA/nBA) copolymer: A – undamaged, A' – Damaged (0 min); 60/40 p(MMA/nBA) copolymer: B – undamaged, B' – damaged (0 min) 60/40 p(MMA/nBA) copolymer.

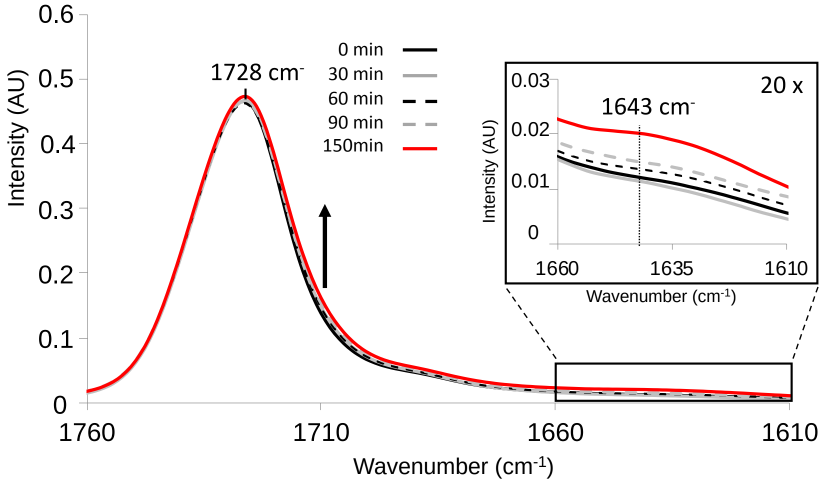

Supplementary Figure 5. ATR FTIR spectra in the carbonyl region of damaged p(MMA/nBA) copolymer exposed to water for 0 (a), 30 (b), 60 (c), 90 (d), 120 (e), and 150 (f) min. The spectra were recorded after exposure to water on dried films. A small fraction of the H_2O-carbonyl H-bonding is detected by slight increase of the C=O band around 1710 cm^{-1} which parallels an increase of the OH bending at 1643 cm^{-1}.

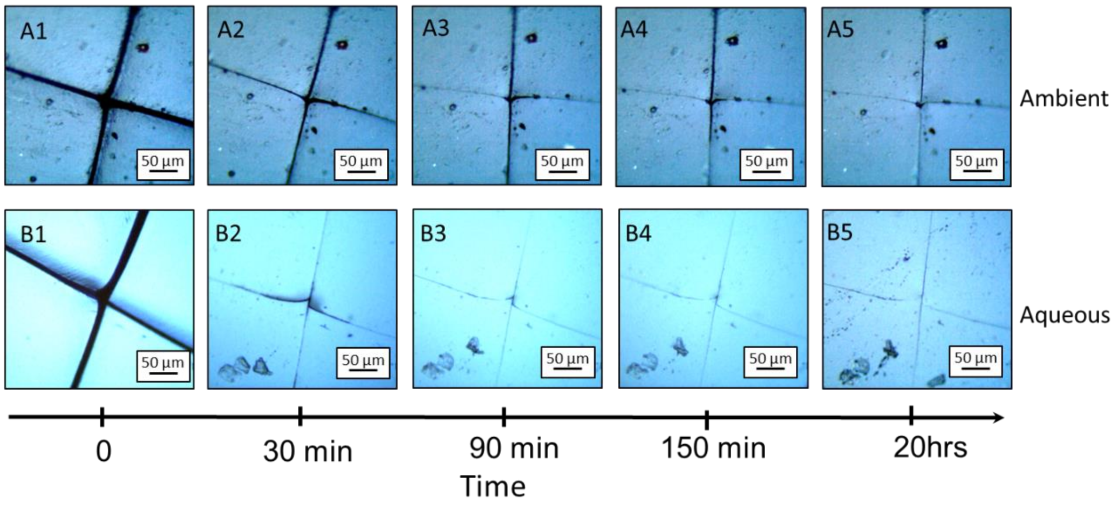

Supplementary Figure 6. Self-healing of air-cut/air-healed (A1-A5) and air-cut/water-healed (B1-B5) 50/50 p(MMA/nPA) copolymers.

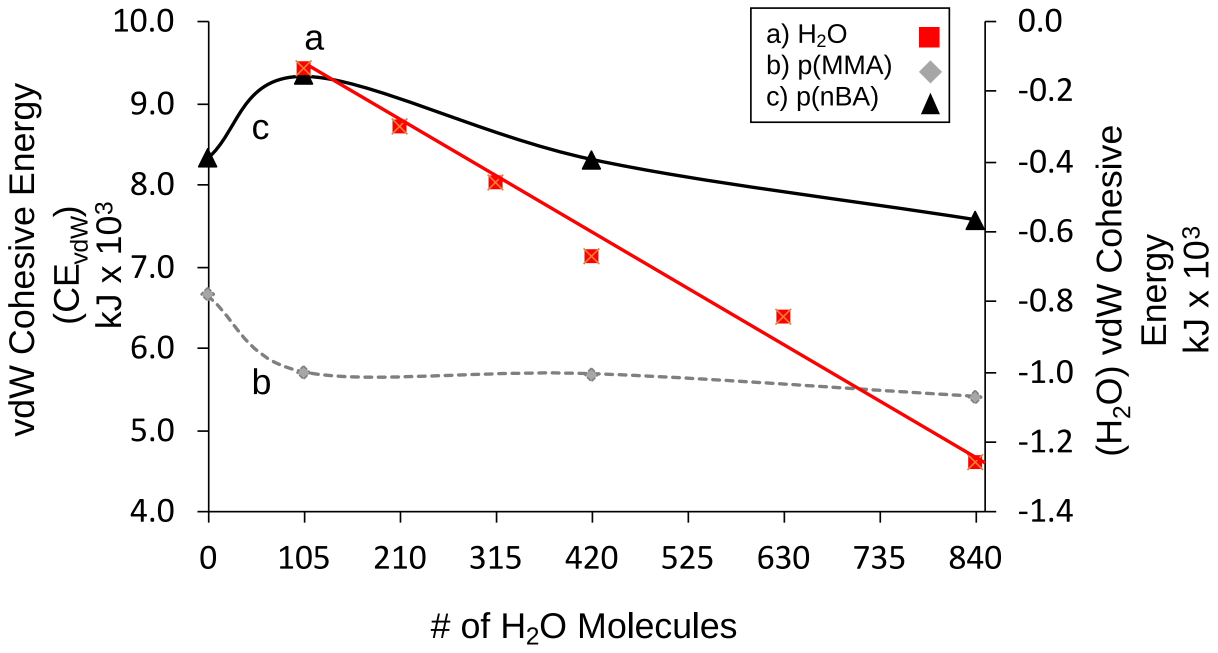

Supplementary Figure 7. Curve a: CE_{vdW} values for H_2O plotted as a function of # H2O molecules (negative values indicate repulsive forces); Curves b and c: CE_{vdW} values plotted as a function of # of H_2O molecules for pMMA (b) and pnBA (c) homopolymers.

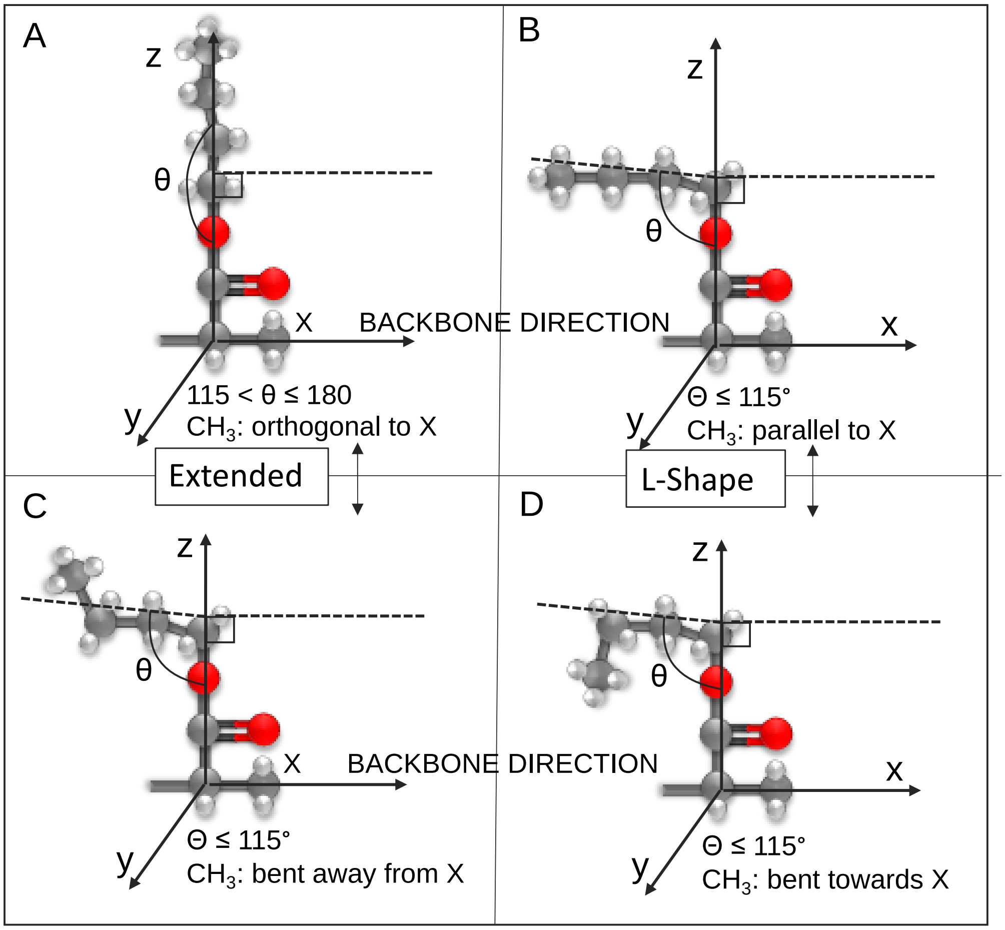

Supplementary Figure 8. nBA side group confirmations: A and C represent extended confirmations, whereas B and D are L-shape (nBA-L).

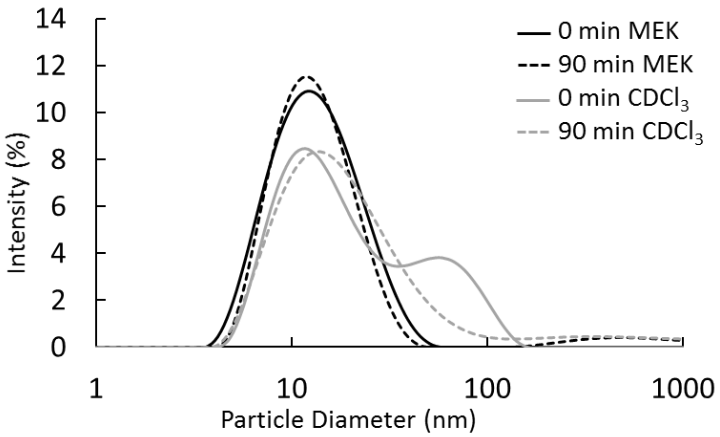

Supplementary Figure 9. p(MMA/nBA) particle size distribution of undamaged vortexed samples in $CDCl_3$ and MEK at 0 and 90 min after initial dissolution.

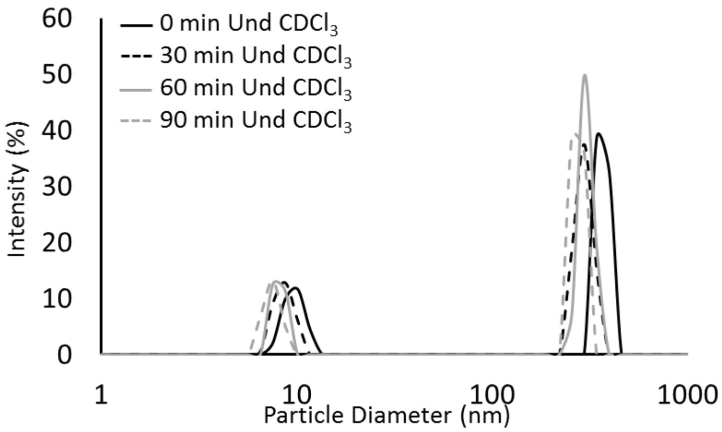

Supplementary Figure 10. p(MMA/nBA) particle size distribution of undamaged non-vortexed samples in $CDCl_3$ at 0, 30, 60 and 90 min after initial dissolution.

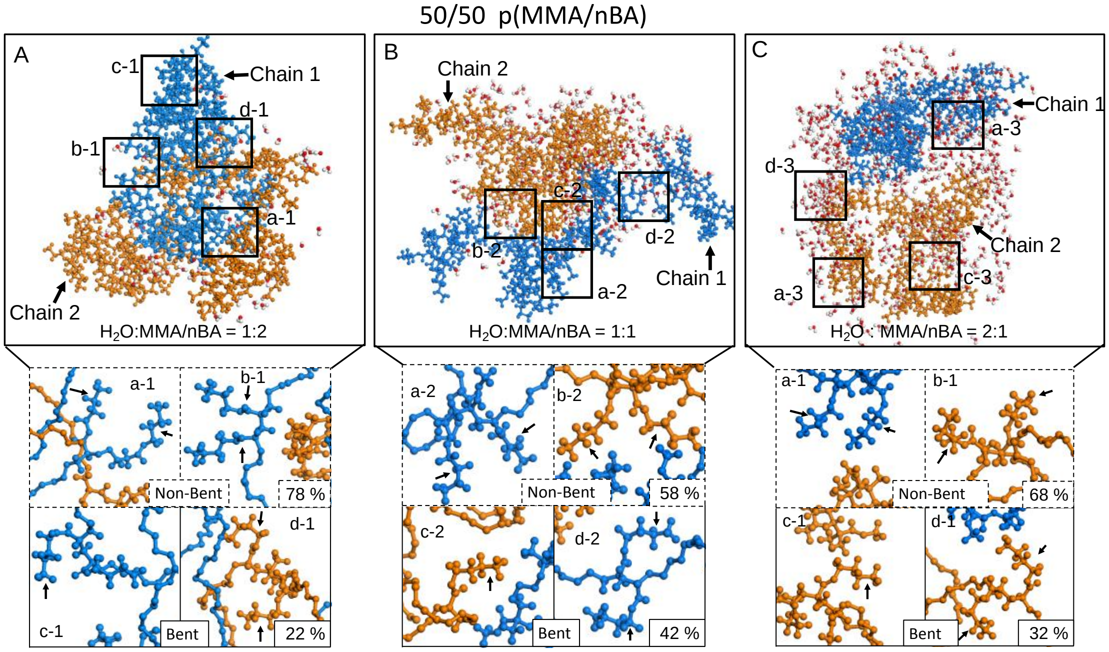

Supplementary Figure 11. Two neighboring p(MMA/nBA) copolymer chains extracted from MD simulations for 50/50 p(MMA/nBA) containing 210 (A1; H_2O:MMA/nBA = 1:2), 420 (A2; H_2O:MMA/nBA = 1:1), and 630 (A3; H_2O:MMA/nBA = 2:1) molecules of H_2O. Squares a-1, b-1, c-1, and d-1 are extracted structural features which show that vdW interactions effectively compete with polymer-water H-bonding when H_2O:MMA/nBA = 1:1.

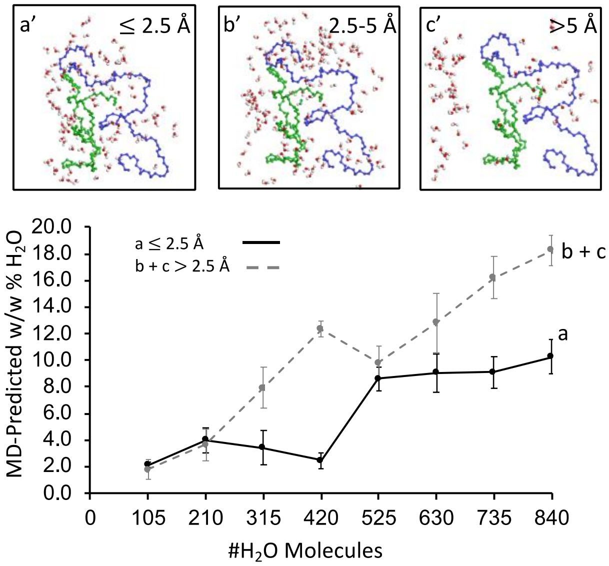

Supplementary Figure 12. Images a', b', and c' (top) represent extracted from MD simulations H_2O molecules within (a') close proximity (≤ 2.5 Å) of two copolymer chains, (b') located between 2.5 - 5 Å from copolymer chains, and (c') located > 5.0 Å from copolymer chains. Supplementary Fig 12, curves a and b + c, illustrate w/w % of H_2O plotted as a function of # H_2O

molecules obtained from MD simulations. These values are also summarized in Supplementary Table 11.

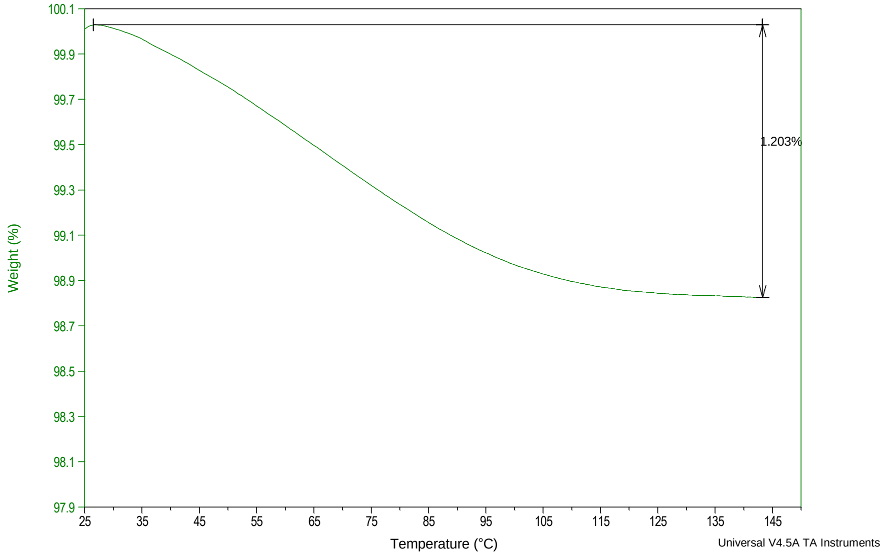

Supplementary Figure 13. Representative TGA curve for p(MMA/nBA) copolymer immersed in H_2O for 24 hrs prior analysis.

Supplementary Tables

Copolymer	MMA/nBA Feed Ratio (f)	MMA/nBA Actual Ratio (F)* (+/-1)	M_n (kDa)	M_w (kDa)	Dispersity (Đ)	T_g (K)	Self-Healing
p(MMA/nBA)	60/40	63/47	54	108	2.00	296	-
	50/50	49/51	39	89	2.28	280	+
	40/60	42/58	49	94	1.93	270	-
p(MMA/nPA)	50/50	54/46	73	141	1.94	280	+

* - determined by ^{1}H NMR spectroscopy; "+" self-healable; "-" no self-healable.

Supplementary Table 1. Number average molecular weight (M_n), weight average molecular weight (M_w), dispersity (Đ), glass transitions (T_g) and self-healability of 63/47, 49/51 and 42/58 p(MMA/nBA) and 54/46 p(MMA/nPA) copolymers.

Time (min)	Condition	Max Strain (%)	Stress at Break (MPa)
0	Undamaged	1221 ± 151	2.41 ± 0.34
0	Air-cut/Air-Healed	540 ± 300	1.30 ± 0.39
30	Air-cut/Air-Healed	564 ± 54	1.67 ± 0.24
30	Air-cut/Water-Healed	986 ± 114	1.90 ± 0.20
90	Air-cut/Air-Healed	698 ± 248	1.75 ± 0.52
90	Air-cut/Water-Healed	845 ± 49	2.27 ± 0.14
150	Air-cut/Air-Healed	1018 ± 218	2.11 ± 0.34
150	Air-cut/Water-Healed	1249 ± 224	2.40 ± 0.27
1200	Air-cut/Air-Healed	882 ± 141	2.19 ± 0.19
1200	Air-cut/Water-Healed	933 ± 185	1.95 ± 0.19

Supplementary Table 2. Maximum strain and stress at break for air-cut/air-healed and air-cut/water-healed 49/51 p(MMA/nBA) copolymers for 30, 90, 150 min and 20 hrs.

Time (min)	p(MMA/nBA)	CH_{3b} - $CH2_{2b}$ Intensity	SNR	Noise Intensity
0	Undamaged-Air	0.59	158	0.0037
0	Air-Cut/Air-Healed	0.71	186	0.0035
60	Air-Cut/Air-Healed	0.64	240	0.0028
60	Air-Cut/Water-Healed	0.52	134	0.0037
180	Air-Cut/Air-Healed	0.54	152	0.0034
180	Air-Cut/Water-Healed	0.48	156	0.0031
19 hrs	Undamaged-Water	0.56	122	0.0037

Supplementary Table 3. ^{1}H NMR of CH_{3b}-CH_{2b} resonance intensities of 50/50 p(MMA/nBA) as a function of exposure to water, signal to noise ratio (SNR), and the noise intensity levels of 49/51 p(MMA/nBA) copolymer.

2D ^{1}H NMR INTENSITIES				
	CH_{3b}-CH_{2b} (a’, a”)		CH_{3m}-CH_b (b’, b”)	
Figure	a’	a”	b’	b”
Fig. 2B	-0.59	-0.86	3.62	3.77
Fig. 2B1	-0.63	-0.96	3.21	3.29
Fig. 2C	-0.70	-1.08	4.11	4.17
Fig. 2C1	-0.53	-1.04	3.59	3.82
Supplementary Figure 1A	-0.59	-0.86	3.62	3.77
Supplementary Figure 1B	na	na	na	na
Supplementary Figure 3A	-0.57	-0.85	2.74	2.82
Supplementary Figure 3B	-0.51	-0.66	2.80	3.02
Supplementary Figure 3B	-0.41	-0.70	2.65	2.90
Supplementary Figure 4A	-2.39	-3.56	11.26	11.48
Supplementary Figure 4A’	-1.57	-1.98	9.36	9.44
Supplementary Figure 4B	0	-0.18	0.48	0.49
Supplementary Figure 4B’	-0.02	-0.04	0.27	0.32

Supplementary Table 4. ^{1}H NMR intensities of resonances due to through-space CH_{3B}-CH_{2B} (a’, a”) and CH_{3m}-CH_B (b’, b”) interactions.

Time (min)	$\chi_{Free\ H2O}$	$\chi_{Dimer\ H2O}$	$\chi_{Small\ cluster\ H2O}$	$\chi_{Large\ cluster\ H2O}$
0	0.38	0.32	0.17	0.12
30	0.32	0.27	0.21	0.19
60	0.32	0.29	0.20	0.18
90	0.29	0.29	0.24	0.19
120	0.23	0.27	0.26	0.24
150	0.21	0.26	0.29	0.24

Supplementary Table 5. The fractions of free water ($\chi_{Free\ H2O}$ 3619 cm^{-1}), dimer water ($\chi_{Dimer\ H2O}$ 3546 cm^{-1}), small water cluster ($\chi_{Small\ cluster\ H2O}$ - 3342 cm^{-1}) and large water clusters ($\chi_{Large\ cluster\ H2O}$ - 3282 cm^{-1}).

# of H_2O molecules	R_w	CE_{total} kJ x 10^3	CE_{vdw} kJ x 10^3	CE_H kJ x 10^3	CED_{total} kJ x $10^5/m^3$	CED_{vdW} kJ x $10^5/m^3$	CED_H kJ x $10^5/m^3$	R_g(Å)	r_{eq}(Å)
0	0:1	6.61	6.61	0.00	1.58	1.58	0	13.38	29.80
105	1:4	8.14	7.11	1.03	1.83	1.70	0.13	15.22	29.13
210	1:2	8.82	6.62	2.20	1.91	1.60	0.31	12.85	28.49
315	3:4	9.45	7.09	2.36	1.98	1.43	0.55	14.42	31.29
420	1:1	10.25	6.81	3.44	2.07	1.36	0.71	13.9	27.59
525	5:4	11.81	6.94	4.87	2.36	1.39	0.97	12.93	27.78
630	3:2	12.54	5.43	7.11	2.38	1.25	1.13	14.57	35.90
735	7:4	13.32	6.92	6.40	2.45	1.23	1.22	13.64	27.12
840	2:1	14.60	6.48	8.12	2.61	1.13	1.48	14.73	29.90

Supplementary Table 6. Total cohesive energy (CE_{total}), van der Waals cohesive energy (CE_{vdW}), hydrogen bonding cohesive energy (CE_H), total cohesive energy density (CED_{total}), van der Waals cohesive energy density (CED_{vdW}), hydrogen bonding cohesive energy density (CED_H), radius of gyration (R_g), and average chain end-to-end distance (r_{eq}) as a function of the # of H_2O molecules in 60/40 p(MMA/nBA) copolymer (R_w is the ratio of water molecules to the # of MMA/nBA repeat units within the simulated cell).

# of H_2O molecules	R_w	CE_{total} kJ x 10^3	CE_{vdw} kJ x 10^3	CE_H kJ x 10^3	CED_{total} kJ x $10^5/m^3$	CED_{vdW} kJ x $10^5/m^3$	CED_H kJ x $10^5/m^3$	R_g(Å)	r_{eq}(Å)
0	0:1	7.71	7.71	0.00	1.76	1.76	0	14.94	30.04
105	1:4	7.81	7.06	0.75	1.73	1.59	0.14	13.85	31.04
210	1:2	8.74	7.45	1.29	1.86	1.58	0.28	15.08	31.10
315	3:4	9.91	7.28	2.63	2.04	1.55	0.49	14.13	27.72
420	1:1	10.70	6.77	3.93	2.13	1.4	0.73	15.11	31.72
525	5:4	11.28	6.76	4.52	2.17	1.28	0.89	14.05	30.70
630	3:2	12.57	6.71	5.86	2.33	1.25	1.08	14.88	31.15
735	7:4	13.07	6.52	6.55	2.37	1.12	1.25	15.46	33.50
840	2:1	14.36	6.43	7.93	2.52	1.14	1.38	14.51	31.21

Supplementary Table 7. Total cohesive energy (CE_{total}), van der Waals cohesive energy (CE_{vdW}), hydrogen bonding cohesive energy (CE_H), total cohesive energy density (CED_{total}), van der Waals cohesive energy density (CED_{vdW}), hydrogen bonding cohesive energy density (CED_H), radius of gyration (R_g), and average chain end-to-end distance (r_{eq}) as a function of the # of H_2O molecules in 40/60 p(MMA/nBA) copolymer (R_w is the ratio of water molecules to the # of MMA/nBA repeat units within the simulated cell.

p(MMA/nBA) MMA/nBA Molar Ratio	% MMA/nBA Alternating Units	Largest block size	Average Block Size	CE_{vdW} (kJ × 10^3)
40/60	25	5.0	3.8	7.77
50/50	37	5.0	3.6	8.45
60/40	20	10.0	5.0	6.61

Supplementary Table 8. Percentage (%) of MMA/nBA alternating units, largest and average block size, and CE_{vdW} values as a function of MMA/nBA molar ratio in p(MMA/nBA) copolymers.

# H_2O Molecules	$X_{nBA\text{-}Extended}$	$X_{nBA\text{-}L\text{-}Shape}$
0	0.82	0.18
105	0.78	0.22
210	0.78	0.22
315	0.72	0.28
420	0.58	0.42
630	0.81	0.19
840	0.68	0.32

Supplementary Table 9. Molar fractions of extended ($X_{nBA\text{-}Extended}$) and L-shaped ($X_{nBA\text{-}L\text{-}Shape}$) nBA side groups in 50/50 p(MMA/nBA) copolymer chains as a function of # H_2O molecules.

		Particle Size (nm)			
		Time (min)			
		0	30	60	90
Non-Vortexed ($CDCl_3$)	Undamaged	289 ± 33<br>10.5 ± 0.3	240 ± 63<br>9.3 ± 1.9	300 ± 43<br>7.8 ± 2.3	232 ± 59<br>7.0 ± 0.6
	Damaged	Out of Range	Out of Range	Out of Range	Out of Range
Vortexed ($CDCl_3$)	Undamaged	54.8 ± 15.7<br>19.2 ± 3.9	59.0 ± 11.0<br>16.3 ± 4.2	21.2±3.8	19.6 ± 1.8
	Damaged	78.1 ± 48.0<br>12.1 ± 2.8	39.3 ± 3.7*	28.0 ± 3.3*	28.2 ± 5.2*
Vortexed (MEK)	Undamaged	20.0 ± 8.3	14.0 ± 0.3	14.0 ± 0.6	14.0 ± 0.3
	Damaged	20.3 ± 9.3	15.4 ± 1.2	14.4 ± 0.20	17.5 ± 3.7

Supplementary Table 10. Dynamic light scattering (DLS) particle size analysis of damaged and undamaged p(MMA/nBA) copolymers dissolved in $CDCl_3$ and MEK with and without agitation by vertexing. * - indicates broad peaks.

Total H_2O	# H_2O ≤ 2.5 Å	# H_2O 2.5 - 5 Å	# H_2O >5 Å
0	0	0	0
105	57 ± 3	32 ± 9	15 ± 11
210	111 ± 25	69 ± 29	30 ± 4
315	94 ± 34	157 ± 36	64 ± 6
420	67 ± 16	240 ±7	113 ± 9
525	250 ± 23	170 ± 19	104 ± 14
630	265 ± 39	213 ±18	152 ± 43
735	266 ± 33	264 ±11	205 ±32
840	301 ± 34	311 ± 9	235 ± 22

Total H_2O	w/w % H_2O ≤ 2.5 Å	w/w % H_2O 2.5 - 5 Å	w/w % H_2O > 5 Å
0	0	0	0
105	2.1 ± 0.2	1.2 ± 0.3	0.6 ± 0.4
210	4.0 ± 0.9	2.5±1.1	1.1 ±0.2
315	3.4 ± 1.3	5.6 ± 1.3	2.4 ± 0.2
420	2.5 ±0.6	8.3 ± 0.2	4.1 ± 0.3
525	8.6 ± 0.9	6.0 ± 0.7	3.8 ± 0.5
630	9.1 ± 1.4	7.4 ± 0.7	5.4 ± 1.6
735	9.1 ± 1.2	7.2±0.4	9.0 ± 1.2
840	10.3 ± 1.3	10.5 ± 0.3	7.8 ± 0.8

Supplementary Table 11. Summary of MD-predicted total # of H_2O molecules, distances, and H_2O w/w % located ≤ 2.5, 2.5 – 5, and 5 Å from p(MMA/nBA) chains.

Time (min)	OH Band Intensity (A.U.) (1643 cm^{-1})	C=O Band Intensity (A.U.) (1726 cm^{-1})	[OH] (mol/L)	[C=O] (mol/L) x 10^{-3}	Exp. H_2O w/w %
0	7.00 10^{-04}	0.484	$3.21x10^{-05}$	$1.61x10^{-3}$	0
30	7.11x 10^{-04}	0.483	$3.26x10^{-05}$	$1.61x10^{-3}$	0.005
60	$1.01x10^{-03}$	0.483	$4.61x10^{-05}$	$1.61x10^{-3}$	0.14
90	$1.28x10^{-03}$	0.482	$5.85x10^{-05}$	$1.61x10^{-3}$	0.27
120	$2.19x10^{-03}$	0.473	$1.01x10^{-04}$	$1.58x10^{-3}$	0.76
150	$2.94x10^{-03}$	0.485	$1.35x10^{-04}$	$1.62x10^{-3}$	1.11
1440	2.78 $x10^{-03}$	0.472	$1.27x10^{-04}$	$1.32x10^{-3}$	1.68

Supplementary Table 12. OH and C=O band intensities, relative concentrations, and experimentally determined w/w % H_2O as a function of time exposure to water.

Time (min)	% Weight Loss of H_2O
30	0.41 ± 0.03
60	0.42 ± 0.02
90	0.45 ± 0.05
120	0.49 ± 0.05
150	0.55 ± 0.08
1440 (24 hrs)	1.17 ± 0.06

Supplementary Table 13. Summary of thermogravimetric analysis (TGA): time exposure of p(MMA/nBA) films to H_2O and the weight loss (%) due to H_2O release. Each % weight loss represents an average of three runs.

Supplementary References

1. M. W. Urban *et al.*, Key-and-lock commodity self-healing copolymers. *Science* **362**, 220-225 (2018).
2. F. C. Schilling, F. A. Bovey, M. D. Bruch, S. A. Kozlowski, Observation of the stereochemical configuration of poly (methyl methacrylate) by proton two-dimensional J-correlated and NOE-correlated NMR spectroscopy. *Macromolecules* **18**, 1418-1422 (1985).
3. A. Aerdts, J. De Haan, A. German, G. Van der Velden, Characterization of intramolecular microstructure of styrene-methyl methacrylate copolymers: new proton NMR assignments supported by 2D-NOESY NMR. *Macromolecules* **24**, 1473-1479 (1991).
4. E. Crispim, I. Schuquel, A. Rubira, E. Muniz, Solvent effects on the miscibility of PMMA/PVAc blends: II. Using two-dimensional NMR method, NOESY. *Polymer* **41**, 933-945 (2000).
5. S. Y. Venyaminov, F. G. Prendergast, Water (H2O and D2O) Molar Absorptivity in the 1000–4000 cm− 1Range and Quantitative Infrared Spectroscopy of Aqueous Solutions. *Analytical biochemistry* **248**, 234-245 (1997).
6. L. Cross, A. Rolfe, Molar extinction coefficients of certain functional groupings with special reference to compounds containing carbonyl. *Transactions of the Faraday Society* **47**, 354-357 (1951).
7. M. Urban, ATR Spectroscopy of Polymers-Theory and Practice. *ACS, Washington DC*, (1996).
8. M. W. Urban, Vibrational spectroscopy of molecules and macromolecules on surfaces. (1993).
